# Surgical treatment of giant mesenteric fibromatosis presenting as a gastrointestinal stromal tumor: a case report

**DOI:** 10.1186/1752-1947-4-314

**Published:** 2010-09-23

**Authors:** Christos N Stoidis, Basileios G Spyropoulos, Evangelos P Misiakos, Christos K Fountzilas, Panorea P Paraskeva, Constantine I Fotiadis

**Affiliations:** 1 Department of Surgery, Athens Navy Hospital, 70 Deinokratous Street, 11521, Athens, Greece; 2 Third Department of Surgery, University of Athens Medical School, Attikon University Hospital, 1 Rimini Street, 12462, Chaidari, Greece; 3 Department of Internal Medicine, Athens Navy Hospital, 70 Deinokratous Street, 11521, Athens, Greece; 4 Second Department of Propaedeutic Surgery, University of Athens Medical School, Laikon General Hospital, 17 Agiou Thoma Street, 11527, Athens, Greece

## Abstract

**Introduction:**

Intra-abdominal fibromatosis, usually located at the mesenteric level, is a locally invasive tumor of fibrous origin, with no ability to metastasize, but a tendency to recur. Certain non-typical cases of intra-abdominal fibromatosis with involvement of the bowel wall can be misdiagnosed because of their different biological behavior.

**Case presentation:**

We describe the case of a 64-year-old Caucasian man presenting with mesenteric fibromatosis and involvement of the bowel wall, who was treated surgically. The macroscopic and microscopic appearance of the lesion mimicked a gastrointestinal stromal tumor, a tumor with potential malignant behavior.

**Conclusion:**

It is essential to make an early and correct diagnosis in such equivocal cases, so that the appropriate treatment can be chosen and suitable patients admitted to clinical trials if appropriate. New and reliable criteria for discriminating between intra-abdominal fibromatosis and gastrointestinal stromal tumor should be proposed and established because novel sophisticated therapeutic strategies have been introduced in the international literature.

## Introduction

Mesenteric desmoids account for less than 10% of sporadic desmoid tumors and are particularly common in patients with familial adenomatous polyposis (FAP) [1]. Of these tumors, 70% are intra-abdominal, and most of these involve the mesentery [1]. The association between desmoid tumors and FAP is particularly strong in patients with Gardner's syndrome [2]. Patients with FAP and a family history of desmoid tumors have a 25% chance of developing a desmoid tumor [2].

Gastrointestinal stromal tumors (GISTs) on the other hand originate from gastrointestinal pacemaker Cajal cells, which are the primary effectors controlling gut motility [3]. GISTs may develop to a large size and usually present with bleeding. Radiologically, the tumor usually contains a central ulceration caused by necrosis from outgrowth of its blood supply. GISTs may grow into the organ lumen, remain entirely on the serosal surface, or even become pedunculated within the abdominal cavity. Spread is by direct invasion or blood-borne metastases. Computed tomography (CT) scans provide useful information about the extent of extra-organ spread.

The enigmatic biology and anatomic location of intra-abdominal fibromatosis (IAF) highlight the need to discriminate between these two diseases. IAF is benign and exclusively locally aggressive, whereas GISTs present with a risk of aggressive clinical behavior, depending on location, diameter and number of mitoses; thus GISTs are potentially malignant and may lead to distant metastases. The fact that IAF and GISTs have different biological behaviors makes treatment recommendations difficult despite the recent introduction of new therapeutic strategies. A significant factor limiting any attempts at generalizing management strategies is the small number of cases available for analysis, reflecting the relative rarity of the disease.

## Case presentation

A 64-year-old Caucasian man was admitted to our hospital with a ten-year-history of a mild diffuse abdominal pain associated with anorexia. He reported no noticeable weight loss or other symptoms. His family history was unremarkable and he had no history of previous abdominal surgery.

On physical examination, we noted a mass in the left upper quadrant of the abdomen, which was mobile. A CT scan of the abdomen revealed a homogeneous, non-enhancing mass, 70 × 100 mm in size, in the mesenteric region near the small bowel, with a consistency suggesting thick mucinous or proteinaceous material, which possibly represented an intestinal wall tumor. There were no other relevant findings.

**Figure 1:**
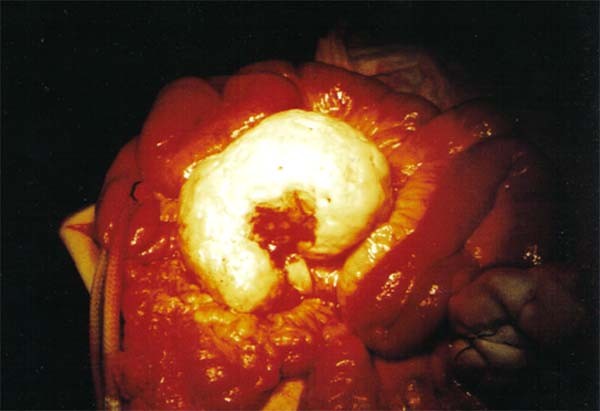
**Surgical preparation**. The resected tumor measured 120 × 80 × 100 mm.

At laparotomy, a solid mass measuring 80 × 100 × 120 mm was identified at the root of the jejunal mesentery, infiltrating the adipose tissue and bowel wall, and in close association with the superior mesenteric and the middle colic vessels (Figure 1). The mesentery contained several large lymph nodes. A small amount of free peritoneal fluid was present. The mass was totally excised with a loop of jejunum and without apparent interference with the blood supply to the bowel. However, bowel ischemia did occur, and the patient required a second laparotomy three days later. The ischemic injury seemed to be secondary to venous obstruction. It was necessary to resect an additional segment of small bowel measuring 600 mm in length. A primary anastomosis was performed.

**Figure 2:**
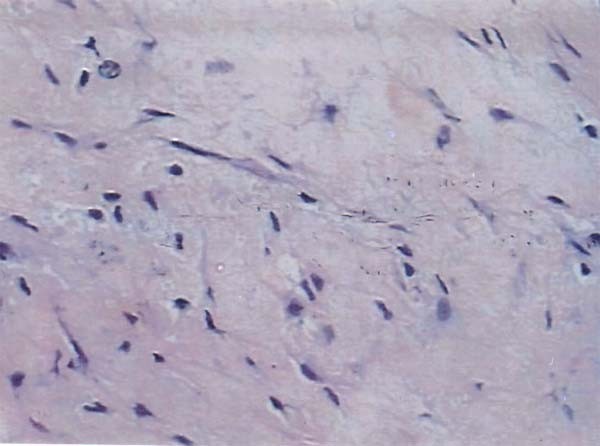
**Spindle cells growing in sweeping fascicles, with eosinophilic cytoplasm**.

Our patient recovered well, and was discharged from the hospital one week after the second laparotomy. The final tissue diagnosis showed spindle-shaped fibroblasts with elongated nuclei and a benign appearance. The cut surface was tan, whorled, and firm, without necrosis, cystic change, or hemorrhage. Microscopy showed loosely arranged spindle cells with bland, oval nuclei and minimal cytoplasm (Figure 2). There were also plump spindle cells with tapering ends, with oval, vesicular nuclei and moderate amounts of eosinophilic cytoplasm. There were many thin-walled vessels of varying caliber. There were no cells with epithelioid features, and any inflammatory cells, calcification, osseous metaplasia, necrosis, or mitoses. The tumor had infiltrated the muscularis propria and had non-infiltrating margins. The tumor cells were negative for antibodies to CD117, S100, CD34, and smooth muscle actin, and positive for desmin. The small sample of peritoneal fluid was free of malignant cells.

A diagnosis of fibromatosis of the jejunum and mesentery was made. Six months after resection, a CT scan of the abdomen showed no evidence of residual or recurrent tumor. Our patient had no evidence of disease at follow-up 16 months after surgery. However, the clinical data and radiologic findings gave rise to a diagnostic dilemma: was the lesion an intermediate or high-risk malignant GIST originating from the bowel wall and spreading to the mesentery, or was this a benign lesion such as IAF originating from the mesentery and infiltrating the bowel wall?

## Discussion

Intra-abdominal spindle cell lesions are uncommon, and often present a diagnostic challenge [4]. The differential diagnosis of a bland spindle cell tumor involving the gastrointestinal (GI) tract and the mesentery includes GIST, fibromatosis, and inflammatory myofibroblastic tumor [5].

IAF is the most common primary mesenteric tumor with spindle cell morphology [6]. Its biological behavior is intermediate between benign fibrous tissue proliferation and fibrosarcoma. Fibromatosis characteristically is locally invasive and tends to recur, but does not metastasize. Although mesenteric desmoid tumors tend to be aggressive, there is considerable variability in their growth rate during the course of the disease. In fact, the biology of intra-abdominal desmoid may be characterized by initial rapid growth, followed by stability or even regression. However, mesenteric desmoid, by virtue of its relationship to vital structures and its ability to infiltrate adjacent organs, may cause important complications, including intestinal obstruction, ischemia and perforation, hydronephrosis, and even aortic rupture [7,8]. Despite these complications, the overall ten-year-survival for patients with intra-abdominal desmoids can be as high as 60 to 70% [9].

In contrast to abdominal wall desmoids, surgery for intra-abdominal desmoid tumors is much more dangerous, and is associated with increased morbidity and mortality, mainly due to hemorrhagic complications or extensive enterectomy (caused by small bowel involvement or involvement of the base of the mesentery and major portions of the mesenteric blood supply) requiring long-term parenteral nutrition [10]. Additionally, a large percentage of patients with intra-abdominal desmoid tumors have unresectable disease [11]. In these cases, expectant treatment is favored, and biopsy should be preferred to excision. If clinical obstruction warrants operative therapy, then bypass, as opposed to major resection, is indicated, followed by a trial of medical therapy. Small bowel obstruction can occur in nearly half of patients with intra-abdominal desmoid tumors, and in 70% of these, diffuse, dense fibrotic adhesions are responsible [12]. Debulking has no place as a therapeutic measure, as it almost invariably leads to more aggressive and infiltrative desmoid growth [12]. Most authors consider surgery a reasonable first-line treatment for abdominal wall tumors, but it should be used as a last resort for intra-abdominal desmoid tumors, and only in specific circumstances (for example, when tumors cause major complications, do not involve vital organs and vessels on preoperative imaging, after failure of systemic pharmacologic therapy, or when surgery is the only possible therapeutic option, such as in the case of a rapidly growing tumor) [13]. Radical (free margin) excision offers the best chance for cure and of avoiding local recurrence [14]. Unfortunately, radical surgery is not always a straightforward procedure because of the extent and invasiveness of the tumor. Therefore, very high rates of recurrence (up to 88%) should be expected [14]. Given the high likelihood of recurrence and prolonged survival even in the setting of advanced disease, some authors have suggested that a trial of watchful waiting along with minimally toxic agents such as sulindac and anti-estrogen therapy may be the best strategy, particularly in patients with minimal symptoms [15]. For clearly inoperable cases, cytotoxic chemotherapy, especially doxorubicin-based or low-dose vinblastin and methotrexate has been proposed [12].

The location of a desmoid tumor within the mesentery of the small bowel may complicate the management of patients with FAP and interfere with surgical strategy, preventing proctectomy or ilial pouch-anal anastomosis (IPAA), at least in some patients [15]. It may also make a diverting ileostomy impossible. Moreover, in this group of patients, recurrence rate after complete resection is particularly high [16]. It has been suggested that this increased risk may be due, at least in part, to the added manipulations of the mesentery during IPAA. Management of desmoid tumors in patients with FAP is further complicated by the fact that clinical course of the disease in this group is particularly variable and unpredictable, and may be disastrous. In a few highly selected patients with extensive desmoid tumors involving the mesentery, intestinal transplantation has been performed [17].

It is generally easy to diagnose primary IAF in its classic presentation as a mesenteric mass, because of its distinctive gross and microscopic features [18]. However, when it presents primarily as an intestinal wall tumor, as in our patient, the diagnosis of GIST must be seriously considered. For our patient, we considered GIST an unlikely diagnosis because of the whorled appearance on the cut surface of the tumor, and the histologic evaluation confirmed this, hence the diagnosis of fibromatosis of the jejunum and mesentery was established.

Histological features characteristic of GIST include the presence of spindle or epithelioid cells with variable architecture, mitotic activity and nuclear atypia, and myxoid or hyalinized stroma. Necrosis and hemorrhage can also be seen. By contrast, IAFs are characterized by a spatially homogeneous proliferation of wavy spindle cells without atypia, associated with collagen deposition (often of the keloidal type) and an infiltrative border. These features are sufficiently characteristic of mesenteric fibromatosis to allow distinction from GIST on the basis of routinely stained sections in most cases. In the few equivocal cases, immunohistochemical analysis can provide more precise diagnosis.

The distinction between these neoplasms is very important because they have different biological behaviors, and there are important clinical implications for the patient. Complete excision of the tumor with a margin of uninvolved tissue (when this is possible) is the most effective treatment yet described, and it may be followed by other therapies if GIST is diagnosed. After the more radical (free margin) resections required for GISTs, the five-year survival rate is approximately 45%, whereas for metastatic disease it drops to 20% [19]. The tumor is resistant to radiotherapy, whereas radiation is controversial for intra-abdominal desmoid tumors [18,19]. Imatinib mesylate is an effective systemic agent and is indicated in patients with KIT (CD117)-positive gastrointestinal stromal tumors that cannot be surgically removed, and/or have spread to other parts of the body. Recent research indicated it may also be useful as part of the post-surgery adjuvant therapy for adult patients who have had their GISTs completely removed. The role of imatinib in the management of abdominal desmoid tumors remains unproven [20].

## Conclusion

The optimum treatment protocol for desmoids tumors has not yet been established and, in many cases, a multidisciplinary approach including surgery, chemotherapy, and radiation therapy is required. The rarity of cases in even major oncological centers has traditionally limited the ability to study this disease. The notion that a specific genotype can predict the development of an aggressive desmoid tumor in a given patient could prove to be valuable in allowing appropriate patient selection for early therapy or even a chemopreventive strategy. Several novel pharmacologic and biologic treatment approaches are actively being developed, although long-term follow-up is needed for their substantiation.

The aim of this report is to stress the importance of correctly characterizing a tumor localized in the bowel wall and infiltrating the mesentery to plan the appropriate treatment because tumor diagnoses based on immunohistochemical staining or traditional histologic criteria alone are not specific enough.

## Consent

Written informed consent was obtained from the patient for publication of this case report and accompanying images. A copy of the written consent is available for review by the Editor-in-Chief of this journal.

## Competing interests

The authors declare that they have no competing interests.

## Authors' contributions

CIF was the patient's surgeon, and was involved in drafting the manuscript and critically revising it for important intellectual content. EPM, CNS, BGS, PPP and CKF have made contributions to conception and design. CNS contributed to the analysis and interpretation of data and wrote the paper. All authors read and approved the final manuscript. All authors contributed equally to the final draft of the manuscript. CIF has given the final approval of the version to be published.
